# 3-Methyl-2-vinyl­pyridinium phosphate

**DOI:** 10.1107/S160053681300576X

**Published:** 2013-03-06

**Authors:** Ganesan Kalaiselvi, Vijayakumar Sabari, Sengottuvelan Balasubramanian, Sanmargam Aravindhan

**Affiliations:** a Department of Inorganic Chemistry, University of Madras, Chennai 600 025, India; b Department of Physics, Presidency College, Chennai 600 005, India

## Abstract

In the title salt, C_8_H_10_N^+^·H_2_PO_4_^−^, the cation is essentially planar (r.m.s. deviation = 0.063 Å). In the crystal, the phosphate anions form inversion *R*_2_^2^(8) dimers *via* pairs of O—H⋯O hydrogen bonds. These dimers are further linked by pairs of O—H⋯O hydrogen bonds, also enclosing *R*_2_^2^(8) loops, forming chains running along [001]. The cations are bonded to the anions *via* N—H⋯O hydrogen bonds and C—H⋯O contacts.

## Related literature

For the biological activity of 4-amino­pyridine, see: Judge & Bever (2006 ▶); Schwid *et al.* (1997 ▶); Strupp *et al.* (2004 ▶). For related structures, see: Anderson *et al.* (2005 ▶); Fun *et al.* (2009 ▶); Sabari *et al.* (2012 ▶).
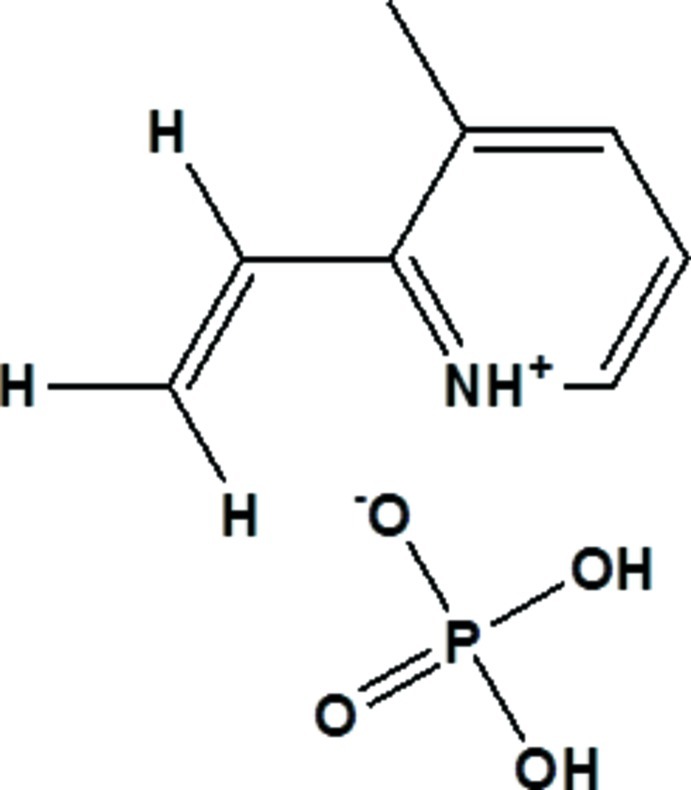


## Experimental

### 

#### Crystal data

C_8_H_10_N^+^·H_2_PO_4_^−^
*M* *_r_* = 217.16
Monoclinic, 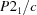

*a* = 7.7089 (6) Å
*b* = 16.3668 (13) Å
*c* = 8.0649 (6) Å
β = 109.689 (4)°
*V* = 958.06 (13) Å^3^
*Z* = 4
Mo *K*α radiation
μ = 0.28 mm^−1^
*T* = 293 K
0.30 × 0.30 × 0.20 mm

#### Data collection

Bruker Kappa APEXII CCD diffractometer
Absorption correction: multi-scan (*SADABS*; Bruker 2008 ▶) *T*_min_ = 0.922, *T*_max_ = 0.947
8970 measured reflections
2362 independent reflections
2052 reflections with *I* > 2σ(*I*)
*R*_int_ = 0.026

#### Refinement

*R*[*F* ^2^ > 2σ(*F* ^2^)] = 0.037
*wR*(*F* ^2^) = 0.114
*S* = 1.08
2362 reflections
136 parameters
3 restraints
H atoms treated by a mixture of independent and constrained refinement
Δρ_max_ = 0.28 e Å^−3^
Δρ_min_ = −0.33 e Å^−3^

### 

Data collection: *APEX2* (Bruker, 2008 ▶); cell refinement: *SAINT* (Bruker, 2008 ▶); data reduction: *SAINT*; program(s) used to solve structure: *SHELXS97* (Sheldrick, 2008 ▶); program(s) used to refine structure: *SHELXL97* (Sheldrick, 2008 ▶); molecular graphics: *ORTEP-3 for Windows* (Farrugia, 2012 ▶); software used to prepare material for publication: *SHELXL97* and *PLATON* (Spek, 2009 ▶).

## Supplementary Material

Crystal structure: contains datablock(s) I, global. DOI: 10.1107/S160053681300576X/bt6887sup1.cif

Structure factors: contains datablock(s) I. DOI: 10.1107/S160053681300576X/bt6887Isup2.hkl

Supplementary material file. DOI: 10.1107/S160053681300576X/bt6887Isup3.cml

Additional supplementary materials:  crystallographic information; 3D view; checkCIF report

## Figures and Tables

**Table 1:** Hydrogen-bond geometry (Å, °)

*D*—H⋯*A*	*D*—H	H⋯*A*	*D*⋯*A*	*D*—H⋯*A*
N1—H1⋯O2	0.86	1.83	2.6558 (18)	160
O3—H3*A*⋯O1^i^	0.89 (1)	1.72 (1)	2.5995 (18)	173 (3)
O4—H4*A*⋯O2^ii^	0.89 (1)	1.72 (1)	2.6002 (17)	170 (3)
C1—H1*A*⋯O1^iii^	0.93	2.48	3.172 (2)	131

Symmetry codes: (i) 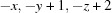; (ii) 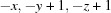; (iii) 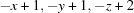.

**Fig. 1.**
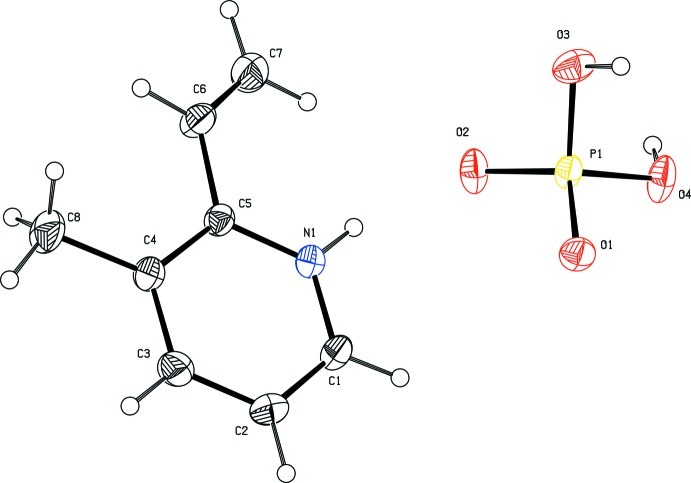
View of one molecule of the title compound showing the atom-labelling scheme. Displacement ellipsoids are drawn at the 30% probability level (arbitrary spheres for the H atoms).

**Fig. 2.**
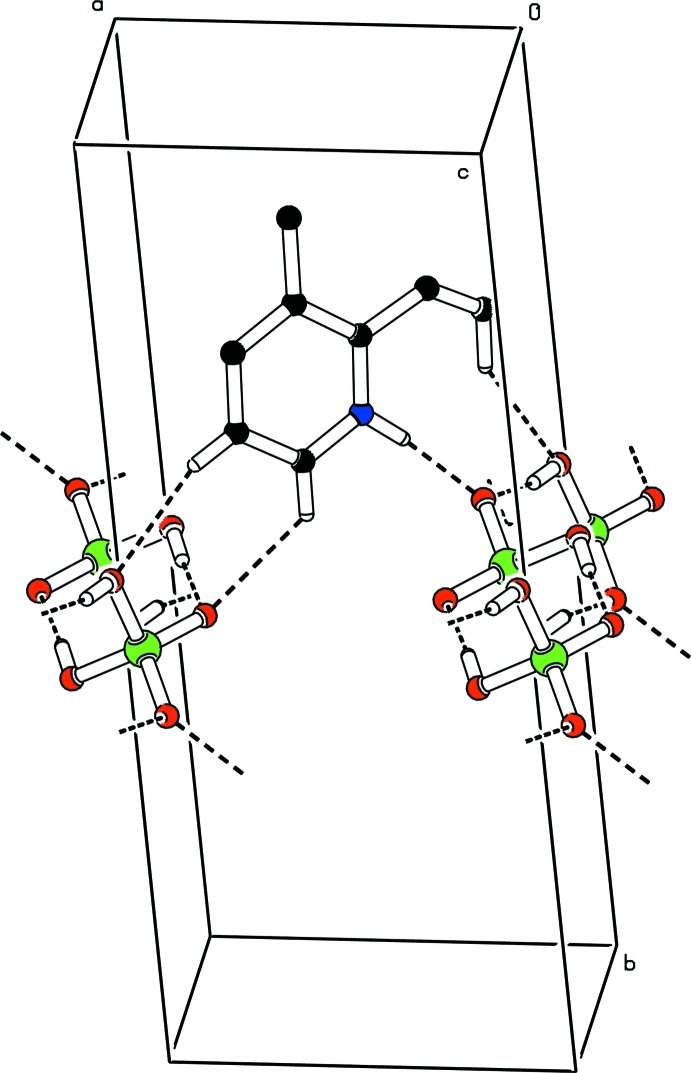
A view of the crystal packing. Hydrogen bonds are drawn as dashed lines. H atoms not involved in hydrogen bonding have been omitted for clarity.

## supplementary crystallographic information

### Comment

4-Aminopyridine (Fampridine) is used clinically in Lambert-Eaton myastheni syndrome and multiple sclerosis because by blocking potassium channels it prolongs action potentials thereby increasing transmitter release at the neuromuscular junction (Judge & Bever *et al.*, 2006; Schwid *et al.*, 1997; Strupp *et al.*, 2004).

In the title compound (Fig. 1), the bond lengths and angles have normal values. The asymmetric unit is composed of one 3-methyl 2-vinyl pyridinium cation and one phosphate anion. The C1—N1—C5 angle in the pyridinium ring is widened to 123.35 (2) °, compared to 115.25 (13)° in 4-aminopyridine (Anderson *et al.*, 2005), 121.20 (15) in 1-(2-carboxyethyl)-5-ethyl-2-methylpyridinium (Sabari *et al.*, 2012) and 120.7 (2)° in Aminopyridinium (Fun *et al.*, 2009). The 3-methyl 2-vinyl pyridinium ring is essentially planar with the maximum deviation from planarity being 0.008 (2) Å for atom C5. The sum of the bond angles around the N1 atom (359.89°) indicates *sp*^2^ hybridization.

The phosphate anions form centrosymmetric *R*_2_^2^(8) dimers *via* O—H···O hydrogen bonds. These dimers are further linked to chains running along the c axis. The cations are boned to the anions via N—H···O hydrogen bonds and C—H···O contacts.

### Experimental

1 g (0.0084 mol) of freshly distilled 3-methyl 2-vinyl pyridine was dissolved in 15 ml of diethyl ether at -10°C under nitrogen atmosphere. To the above solution, 0.5 ml of H_3_PO_4_ and 10 ml of diethyl ether mixture was added in drops with continuous stirring. The product obtained as a white solid was filtered, washed with diethyl ether and dried under vacuum, the product was recrystallized from methanol. Yield: 100% (1.82 g).

### Refinement

All H atoms were found in a difference Fourier map and those bonded to O were refined with a distance restraint of 0.90 (1)Å. The other H atoms were fixed geometrically and allowed to ride on their attached atoms, with C—H ranging from 0.93 to 0.96 Å, and with *U*_iso_ = 1.2–1.5U_eq_ (C,N). The methyl group was allowed to rotate but not to tip.

### Crystal data

**Table:**

C_8_H_10_N^+^·H_2_O_4_P^−^	*F*(000) = 456
*M**_r_* = 217.16	*D*_x_ = 1.506 Mg m^−^^3^
Monoclinic, *P*2_1_/*c*	Mo *K*α radiation, λ = 0.71073 Å
Hall symbol: -P 2ybc	Cell parameters from 8834 reflections
*a* = 7.7089 (6) Å	θ = 2.1–31.2°
*b* = 16.3668 (13) Å	µ = 0.28 mm^−^^1^
*c* = 8.0649 (6) Å	*T* = 293 K
β = 109.689 (4)°	Block, colourless
*V* = 958.06 (13) Å^3^	0.30 × 0.30 × 0.20 mm
*Z* = 4	

### Data collection

**Table:**

Bruker Kappa APEXII CCD diffractometer	2362 independent reflections
Radiation source: fine-focus sealed tube	2052 reflections with *I* > 2σ(*I*)
Graphite monochromator	*R*_int_ = 0.026
ω and φ scan	θ_max_ = 28.3°, θ_min_ = 2.5°
Absorption correction: multi-scan (*SADABS*; Bruker 2008)	*h* = −10→10
*T*_min_ = 0.922, *T*_max_ = 0.947	*k* = −21→17
8970 measured reflections	*l* = −10→10

### Refinement

**Table:**

Refinement on *F*^2^	Primary atom site location: structure-invariant direct methods
Least-squares matrix: full	Secondary atom site location: difference Fourier map
*R*[*F*^2^ > 2σ(*F*^2^)] = 0.037	Hydrogen site location: inferred from neighbouring sites
*wR*(*F*^2^) = 0.114	H atoms treated by a mixture of independent and constrained refinement
*S* = 1.08	*w* = 1/[σ^2^(*F*_o_^2^) + (0.0631*P*)^2^ + 0.2803*P*] where *P* = (*F*_o_^2^ + 2*F*_c_^2^)/3
2362 reflections	(Δ/σ)_max_ = 0.003
136 parameters	Δρ_max_ = 0.28 e Å^−^^3^
3 restraints	Δρ_min_ = −0.33 e Å^−^^3^

### Special details

**Table:**

Geometry. All e.s.d.'s (except the e.s.d. in the dihedral angle between two l.s. planes) are estimated using the full covariance matrix. The cell e.s.d.'s are taken into account individually in the estimation of e.s.d.'s in distances, angles and torsion angles; correlations between e.s.d.'s in cell parameters are only used when they are defined by crystal symmetry. An approximate (isotropic) treatment of cell e.s.d.'s is used for estimating e.s.d.'s involving l.s. planes.
Refinement. Refinement of *F*^2^ against ALL reflections. The weighted *R*-factor *wR* and goodness of fit *S* are based on *F*^2^, conventional *R*-factors *R* are based on *F*, with *F* set to zero for negative *F*^2^. The threshold expression of *F*^2^ > σ(*F*^2^) is used only for calculating *R*-factors(gt) *etc*. and is not relevant to the choice of reflections for refinement. *R*-factors based on *F*^2^ are statistically about twice as large as those based on *F*, and *R*- factors based on ALL data will be even larger.

### Fractional atomic coordinates and isotropic or equivalent isotropic displacement parameters (Å^2^)

**Table:**

	*x*	*y*	*z*	*U*_iso_*/*U*_eq_	
C1	0.5293 (2)	0.35216 (10)	0.9141 (2)	0.0394 (4)	
H1A	0.5318	0.4089	0.9225	0.047*	
C2	0.6707 (3)	0.30687 (12)	1.0253 (3)	0.0446 (4)	
H2	0.7706	0.3322	1.1082	0.054*	
C3	0.6608 (2)	0.22259 (11)	1.0112 (3)	0.0420 (4)	
H3	0.7546	0.1911	1.0870	0.050*	
C4	0.5138 (2)	0.18417 (10)	0.8859 (2)	0.0349 (4)	
C5	0.3751 (2)	0.23315 (9)	0.7738 (2)	0.0304 (3)	
C6	0.2165 (2)	0.19908 (11)	0.6336 (2)	0.0404 (4)	
H6	0.1973	0.1432	0.6382	0.048*	
C7	0.1003 (3)	0.23843 (13)	0.5042 (3)	0.0514 (5)	
H7A	0.1131	0.2945	0.4934	0.062*	
H7B	0.0040	0.2106	0.4221	0.062*	
C8	0.5063 (3)	0.09263 (11)	0.8732 (3)	0.0545 (5)	
H8A	0.3981	0.0732	0.8944	0.082*	
H8B	0.6139	0.0699	0.9594	0.082*	
H8C	0.5020	0.0762	0.7576	0.082*	
N1	0.38850 (18)	0.31502 (8)	0.79424 (17)	0.0319 (3)	
H1	0.3015	0.3448	0.7263	0.038*	
O1	0.20750 (17)	0.49478 (7)	0.95492 (15)	0.0374 (3)	
O2	0.12761 (18)	0.42554 (7)	0.65535 (15)	0.0414 (3)	
O3	−0.11397 (18)	0.44558 (8)	0.78857 (17)	0.0460 (3)	
O4	0.0217 (2)	0.56754 (7)	0.68547 (16)	0.0446 (3)	
P1	0.06935 (6)	0.48168 (2)	0.77599 (5)	0.03024 (15)	
H3A	−0.143 (4)	0.4698 (16)	0.875 (3)	0.082 (9)*	
H4A	−0.032 (4)	0.5639 (19)	0.5694 (14)	0.093 (10)*	

### Atomic displacement parameters (Å^2^)

**Table:**

	*U*^11^	*U*^22^	*U*^33^	*U*^12^	*U*^13^	*U*^23^
C1	0.0427 (9)	0.0273 (8)	0.0485 (10)	−0.0054 (6)	0.0156 (8)	−0.0067 (7)
C2	0.0361 (9)	0.0435 (10)	0.0475 (10)	−0.0078 (7)	0.0052 (8)	−0.0079 (8)
C3	0.0358 (9)	0.0410 (10)	0.0443 (10)	0.0033 (7)	0.0070 (8)	0.0046 (8)
C4	0.0373 (8)	0.0276 (8)	0.0396 (9)	0.0001 (6)	0.0127 (7)	0.0030 (6)
C5	0.0335 (8)	0.0259 (7)	0.0333 (8)	−0.0020 (6)	0.0132 (6)	0.0008 (6)
C6	0.0433 (9)	0.0311 (8)	0.0416 (9)	−0.0076 (7)	0.0075 (8)	−0.0013 (7)
C7	0.0503 (11)	0.0437 (10)	0.0529 (11)	0.0008 (8)	0.0077 (9)	−0.0073 (9)
C8	0.0614 (12)	0.0271 (9)	0.0652 (13)	0.0024 (8)	0.0085 (10)	0.0084 (9)
N1	0.0333 (7)	0.0259 (7)	0.0360 (7)	0.0012 (5)	0.0113 (6)	0.0007 (5)
O1	0.0367 (6)	0.0395 (6)	0.0296 (6)	−0.0004 (5)	0.0028 (5)	−0.0013 (5)
O2	0.0573 (8)	0.0318 (6)	0.0308 (6)	0.0166 (5)	0.0092 (5)	0.0004 (5)
O3	0.0444 (7)	0.0518 (8)	0.0377 (7)	−0.0129 (6)	0.0083 (6)	−0.0120 (6)
O4	0.0703 (9)	0.0243 (6)	0.0330 (6)	0.0110 (5)	0.0093 (6)	0.0008 (5)
P1	0.0375 (2)	0.0230 (2)	0.0260 (2)	0.00340 (14)	0.00509 (17)	−0.00119 (14)

### Geometric parameters (Å, º)

**Table:**

C1—N1	1.333 (2)	C7—H7A	0.9300
C1—C2	1.372 (3)	C7—H7B	0.9300
C1—H1A	0.9300	C8—H8A	0.9600
C2—C3	1.384 (3)	C8—H8B	0.9600
C2—H2	0.9300	C8—H8C	0.9600
C3—C4	1.389 (2)	N1—H1	0.8600
C3—H3	0.9300	O1—P1	1.4923 (12)
C4—C5	1.397 (2)	O2—P1	1.5122 (12)
C4—C8	1.502 (2)	O3—P1	1.5663 (13)
C5—N1	1.350 (2)	O3—H3A	0.889 (10)
C5—C6	1.467 (2)	O4—P1	1.5691 (12)
C6—C7	1.295 (3)	O4—H4A	0.889 (10)
C6—H6	0.9300		
			
N1—C1—C2	120.08 (16)	C6—C7—H7B	120.0
N1—C1—H1A	120.0	H7A—C7—H7B	120.0
C2—C1—H1A	120.0	C4—C8—H8A	109.5
C1—C2—C3	118.37 (17)	C4—C8—H8B	109.5
C1—C2—H2	120.8	H8A—C8—H8B	109.5
C3—C2—H2	120.8	C4—C8—H8C	109.5
C2—C3—C4	121.31 (17)	H8A—C8—H8C	109.5
C2—C3—H3	119.3	H8B—C8—H8C	109.5
C4—C3—H3	119.3	C1—N1—C5	123.48 (14)
C3—C4—C5	118.04 (15)	C1—N1—H1	118.3
C3—C4—C8	120.31 (16)	C5—N1—H1	118.3
C5—C4—C8	121.65 (16)	P1—O3—H3A	110.5 (19)
N1—C5—C4	118.69 (14)	P1—O4—H4A	113 (2)
N1—C5—C6	118.75 (14)	O1—P1—O2	116.08 (7)
C4—C5—C6	122.55 (15)	O1—P1—O3	110.92 (7)
C7—C6—C5	127.06 (17)	O2—P1—O3	106.41 (8)
C7—C6—H6	116.5	O1—P1—O4	107.46 (7)
C5—C6—H6	116.5	O2—P1—O4	108.58 (7)
C6—C7—H7A	120.0	O3—P1—O4	107.04 (8)
			
N1—C1—C2—C3	−1.1 (3)	C8—C4—C5—C6	−1.8 (2)
C1—C2—C3—C4	1.1 (3)	N1—C5—C6—C7	13.1 (3)
C2—C3—C4—C5	0.1 (3)	C4—C5—C6—C7	−166.36 (19)
C2—C3—C4—C8	−179.99 (18)	C2—C1—N1—C5	−0.2 (2)
C3—C4—C5—N1	−1.3 (2)	C4—C5—N1—C1	1.3 (2)
C8—C4—C5—N1	178.81 (16)	C6—C5—N1—C1	−178.12 (15)
C3—C4—C5—C6	178.18 (15)		

### Hydrogen-bond geometry (Å, º)

**Table:**

*D*—H···*A*	*D*—H	H···*A*	*D*···*A*	*D*—H···*A*
N1—H1···O2	0.86	1.83	2.6558 (18)	160
O3—H3*A*···O1^i^	0.89 (1)	1.72 (1)	2.5995 (18)	173 (3)
O4—H4*A*···O2^ii^	0.89 (1)	1.72 (1)	2.6002 (17)	170 (3)
C1—H1*A*···O1^iii^	0.93	2.48	3.172 (2)	131

Symmetry codes: (i) −*x*, −*y*+1, −*z*+2; (ii) −*x*, −*y*+1, −*z*+1; (iii) −*x*+1, −*y*+1, −*z*+2.
